# Characterization of a β-Galactosidase from *Kosakonia oryzendophytica* and Its Heterologous Expression in *Bacillus subtilis* for Galactooligosaccharides Production

**DOI:** 10.3390/molecules30224343

**Published:** 2025-11-10

**Authors:** Zhuo Cheng, Xiangpeng Jin, Yulei Zhang, Dawei Ni, Yingying Zhu, Wei Xu, Wenli Zhang, Wanmeng Mu

**Affiliations:** State Key Laboratory of Food Science and Resources, School of Food Science and Technology, Jiangnan University, Wuxi 214122, China

**Keywords:** galactooligosaccharides, β-galactosidase, hydrolysis, transglycosylation

## Abstract

Galactooligosaccharides (GOS) typically consist of 2-8 D-galactose units linked together, terminating in a D-glucose unit. GOS are commonly used in dairy products, infant formulas, and functional foods. GOS offer beneficial properties for food processing, such as low caloric value, mild clean taste, and excellent solubility in water. Additionally, GOS function as non-digestible prebiotics, supporting microbiota balance and offering benefits such as promoting infant health, immune modulation, laxative effects, and potential metabolic advantages. β-galactosidase plays a key role in GOS production, catalyzing both hydrolysis and transglycosylation reactions. In this study, a putative GH2 family β-galactosidase from *Kosakonia oryzendophytica* (Koor β-gal) was identified. The enzyme exhibited optimal activity at pH 7.0 and 45–50 °C with the addition of 1 mM Mg^2+^, showing a specific activity of approximately 288.6 U/mg towards *o*-nitrophenyl-β-D-galactopyranoside (ONPG). After optimizing the reaction conditions, Koor β-gal successfully produced 124.7 g/L of GOS from 300 g/L D-lactose, achieving a GOS yield of 41.6%. LC-MS analysis revealed that the primary products consisted of GOS with degrees of polymerization (DP) ranging from 2 to 4. Additionally, Koor β-gal was heterologously expressed in *Bacillus subtilis* following comprehensive optimization of the promoter and 5′-UTR, resulting in an enzyme activity in culture filtrate of 106.2 U/mL after 60 h.

## 1. Introduction

D-Lactose, a disaccharide consisting of D-glucose and D-galactose, is abundantly present in dairy-derived products, particularly in cheese whey. Whey lactose has recently gained considerable interest due to its potential for high-value bioconversion [1]. Galactooligosaccharides (GOS) stand out as highly promising D-lactose derivatives, typically produced via enzymatic transgalactosylation using β-galactosidases (β-gal). GOS usually consist of 2-8 D-galactose units, commonly ending in a terminal D-glucose, interconnected through β-(1 → 3), β-(1 → 4), or β-(1 → 6) glycosidic linkages.

GOS are commonly employed as non-digestible prebiotics. They stimulate the growth and adhesion of beneficial bacteria in the gut, and improve the resistance against pathogen colonization, thereby reducing the risk of colon infections. Additionally, GOS can promote the production of short-chain fatty acids (SCFA) and lower colonic pH. These changes are associated with a decreased incidence of colorectal cancer, improved mineral absorption, regulation of blood lipids and cholesterol levels, as well as enhanced intestinal motility and relief of constipation [2]. In addition, the combination of GOS and *Lactobacillus reuteri* as a novel synbiotic could effectively reduce intestinal inflammation and alleviate barrier dysfunction, primarily by enhancing the pentadecanoic acid biosynthesis mediated by *Bacteroides acidifaciens* [3]. Furthermore, the supplementation of GOS and *Lactiplantibacillus plantarum* could improve lipid metabolic dysfunction in obese individuals, primarily by promoting arginine synthesis and stimulating the AMP-activated protein kinase (AMPK) signaling pathway [4].

GOS are commonly added to infant formula, fermented dairy products, baked goods, confectionery, and nutritional supplements. In infant formula, GOS have been extensively applied as a mimic of human milk oligosaccharides (HMOs), promoting the growth of *Bifidobacterium* in neonates [5]. In products like ice cream [6] and yogurt [7], GOS contribute to improved flavor, texture, and mouthfeel, enhancing attributes such as creaminess and smoothness. Additionally, GOS can serve as a sucrose alternative in chocolate, offering the dual benefit of lowering post-meal blood glucose spikes while enhancing the product’s viscosity and hardness [8].

In industrial processes, GOS are manufactured by catalyzing D-lactose as the raw material with β-galactosidases (β-gal). β-Gals (EC 3.2.1.23) catalyze the hydrolysis of lactose into D-glucose and D-galactose, as well as the transgalactosylation reaction forming GOS. β-gals are distributed in different glycoside hydrolase (GH) families in the Carbohydrate-Active Enzymes (CAZy) database, including GH1, GH2, GH35, GH42, GH59, and GH147 [9,10]. β-gals have been isolated and identified from a wide range of microorganisms, including bacteria, yeast, filamentous fungi. The microbial origins of β-gals significantly influence the production of GOS synthesis, such as yield, glycosidic linkage types, and the degree of polymerization. For instance, the β-gal derived from *Bacteroides xylanisolvens* (a gut bacterium) could specifically generate β-1,2-linked GOS, which is distinct from the β-1,3, β-1,4 or β-1,6-linked GOS components produced by most commercially used microbial β-gals [10]. Furthermore, reaction conditions, such as pH, temperature, the β-gal enzyme amount, and substrate concentration, play a crucial role in determining GOS yield [11]. The quest for β-gals with high catalytic activity, excellent thermostability, and superior transglycosylation ratio is vital in GOS biosynthesis. Moreover, for industrial applications, recombinant expression of β-gals is essential, with *Bacillus subtilis* [12] and yeast [13] frequently used as host organisms for large-scale enzyme production.

In this study, a novel β-gal (Koor β-gal) was derived from *Kosakonia oryzendophytica* through in silico screening. The Koor β-gal was initially heterologously expressed in *Escherichia coli*, followed by purification and biochemical characterization. To maximize GOS production, key reaction parameters including temperature, pH, enzyme dosage, and D-lactose concentration were systematically optimized. Furthermore, the expression of Koor β-gal in *B. subtilis* was explored, by comprehensive optimization of the promoters and 5′-UTR.

## 2. Results and Discussion

### 2.1. Amino Acid Sequence and Structure Analysis

**Figure 1:**
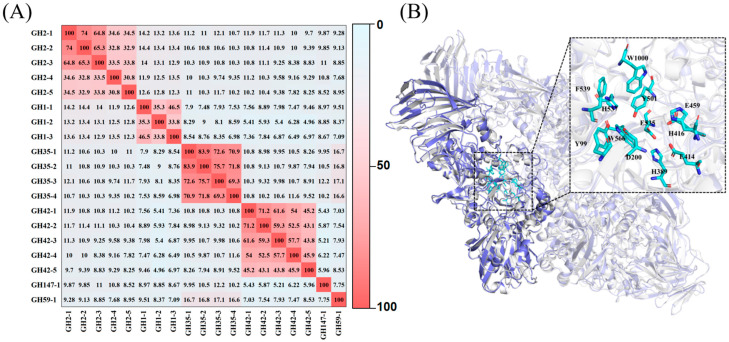
Sequence identity analysis and three-dimensional structural model of Koor β-gal. (**A**) Amino acid sequence identity matrix of Koor β-gal with β-gals from different GH families. The different color in the matrix represents the percentage of sequence identity, ranging from low (light blue) to high (red). (**B**) Superimposition of the predicted three-dimensional structure of Koor β-gal (blue) and the crystal structure of *E. coli* β-gal (gray, PDB: 1JYN). The numerals GH2-1 to GH2-5 indicate that the β-gal belongs to the GH2 family, corresponding to *Kosakonia oryzendophytica* (SCB95762.1), *Enterobacter cloacae* (WFG05994.1), *E. coli* UMN026 (YP_002411150.1), *Lactobacillus delbrueckii* subsp. *Bulgaricus* (ACE06986.1), *Actinobacillus pleuropneumoniae* (AAB17954.1) respectively. GH1-1 to GH1-3 indicate that the β-gal belongs to the GH1 family, corresponding to *Pyrococcus woesei* (AAB97862.1), *Dictyoglomus turgidum* DSM 6724 (ACK43071.1), *Pyrococcus furiosus* (AHW49177.1) respectively. GH35-1 to GH35-4 indicate that the β-gal belongs to the GH35 family, corresponding to *Talaromyces aerugineus* (ADW66246.1), *Penicillium* sp. (CAF32457.1), *Aspergillus tamarii* (KAE8167710.1), *Aspergillus oryzae* (AHI44625.1) respectively. GH42-1 to GH42-5 indicate that the β-gal belongs to the GH42 family, corresponding to *Rahnella* sp. R3 (AJC52391.1), *Klebsiella pneumoniae* (AEA30144.1), *Niallia circulans* (AAA22260.1), *Clostridium perfringens* (BAA08485.1), *Paenibacillus* sp. MDMC362 (WP_113059511.1) respectively. GH147-1 indicate that the β-gal belongs to the GH147 family, corresponding to *Bacteroides ovatus* (ALJ46469.1). GH59-1 indicate that the β-gal belongs to the GH59 family corresponding *Ruminiclostridium cellulolyticum* H10 (ABG76970.1).

The potential β-gal gene from *Kosakonia oryzendophytica* (Koor β-gal) was deposited under GenBank locus_tag FMAY01000003.1. This sequence comprised 3075 bp, encoding 1024 amino acids. Computational analysis with ExPASy predicted Koor β-gal (GenBank accession number: MBH1854985.1) to possess a theoretical molecular weight (Mw) of 116.7 kDa and an isoelectric point (pI) of 5.61. As shown in Figure 1, the sequence identity matrix indicated that Koor β-gal belonged to the GH2 family, sharing more than 34.5% identity with other members, with the highest similarity (74.0%) observed with the β-gal from *Enterobacter cloacae* (WFG05994.1) (GH2-2). The three-dimensional structure of Koor β-gal was modeled using AlphaFold3, with homology to the GH2 β-gal from *E. coli* (PDB: 1JYN). Additionally, crucial residues in the active site of Koor β-gal, such as Y99, D200, H389, E414, H416, E459, Y501, E535, H538, W566, F599, and W1000 were highly conserved.

### 2.2. Biochemical Characterization of Koor β-Gal

**Figure 2:**
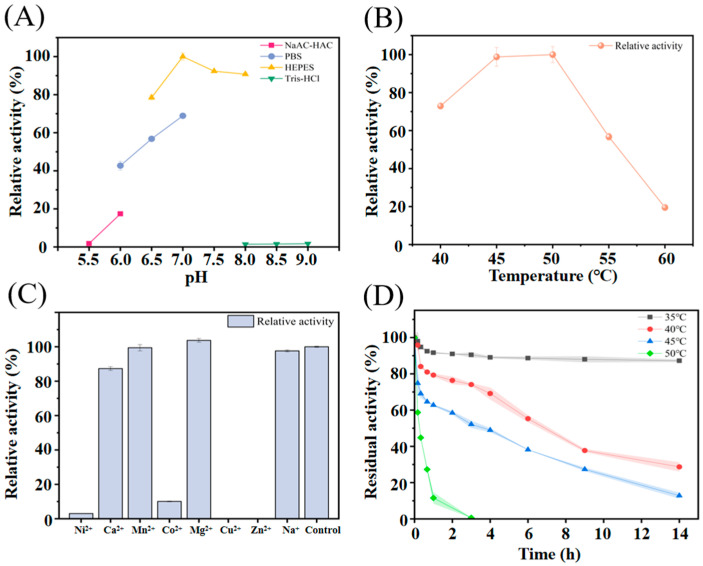
Characterization of Koor β-gal. (**A**) Effect of pH (6.0–8.5) on Koor β-gal activity. The enzyme activity was assayed at 50 °C for 5 min using 15 mM ONPG as the substrate in different buffers (100 mM). (**B**) Effect of temperature (40–60 °C) on Koor β-gal activity. The activity was measured at pH 7.0 (100 mM HEPES buffer) for 5 min with 15 mM ONPG. (**C**) Effect of metal ions on Koor β-gal activity. The relative activity was determined under standard conditions (pH 7.0, 50 °C) in the presence of 1 mM of various metal ions. The activity without any added metal ion was set as 100%. (**D**) Thermostability of Koor β-gal at 35–50 °C The purified enzyme was incubated in 50 mM HEPES buffer (pH 7.0) at the indicated temperatures. Aliquots were withdrawn at specific time intervals, and the residual activity was measured under standard assay conditions (pH 7.0, 50 °C, 15 mM ONPG, 5 min). Error bars represent the standard deviation (SD) of three independent biological replicates.

The enzyme exhibited its highest activity at pH 7.0 in HEPES buffer (Figure 2A), and retained more than 90.7% of its activity under mildly alkaline conditions (pH 7.0–8.0, HEPES buffer). Beyond this range, a decrease in catalytic efficiency below 75% was observed. Moreover, the type of buffer influenced performance notably. For example, at pH 8.0 HEPES buffer, the relative activities were measured as 90.7%. However, at pH 8.0 Tris-HCl buffer, Koor β-gal was almost inactive.

Temperature is a key factor influencing enzymatic performance. To determine the optimal temperature of Koor β-gal, assays were carried out within the range of 40–60 °C under standard reaction conditions (Figure 2B). Koor β-gal retained more than 72.9% of the maximum activity between 40 and 50 °C, reaching the maximum at 45–50 °C. However, when the temperature exceeded 55 °C, activity dropped sharply, falling to only 19.5% at 60 °C.

To investigate the influence of metal ions on Koor β-gal activity, different ions (1 mM each) were supplemented into the reaction mixture, including Na^+^, Zn^2+^, Cu^2+^, Mg^2+^, Co^2+^, Mn^2+^, Ca^2+^, and Ni^2+^ (Figure 2C). The presence of Mn^2+^, Na^+^ and Ca^2+^ resulted in slight decreases, with relative activities of 99.4% and 87.2%, respectively. In comparison, Zn^2+^, Cu^2+^, Co^2+^, and Ni^2+^ caused a near-complete inactivation of the enzyme.

The thermostability of Koor β-gal was assessed across 35–50 °C (Figure 2D). At 35 °C, the enzyme remained highly stable, showing almost no loss of activity even after 14 h of incubation. In contrast, exposure to 50 °C drastically reduced stability, with nearly complete inactivation observed within 3 h. The half-life (*t*_1/2_) values of Koor β-gal were calculated to be 7.4 and 3.5 h at 40 and 45 °C, respectively. Previous studies have demonstrated that metal ions can improve not only enzymatic activity but also thermostability [14]. Nevertheless, for Koor β-gal, the addition of metal ions (Mg^2+^) had little effect on *t*_1/2_ value. The corresponding data is provided in Supplementary Figure S3. In the context of industrial GOS production, β-gals with strong thermostability at elevated temperatures are preferred, as they promote improving D-lactose solubility, reduce system viscosity, transgalactosylation efficiency, and help suppress microbial contamination [2].

The kinetic characteristics of Koor β-gal were evaluated using ONPG as the substrate at concentrations ranging from 0.3 to 50 mM (Figure S4). The Michaelis constant (*K*_m_), catalytic turnover rate (*k*_cat_), and catalytic efficiency (*k*_cat_/*K*_m_) were determined to be 6.0 ± 0.3 mM, 732.7 ± 43.5 s^−1^, and 122.1 ± 13.4 mM^−1^·s^−1^, respectively. Under the optimal conditions of pH 7.0 and 45–50 °C, Koor β-gal demonstrated a maximum specific activity of 288.6 U/mg toward ONPG. Compared with other previously reported β-gals from different sources, the *K*_m_ value of Koor β-gal for ONPG is 6.0 ± 0.3 mM, which is higher than that of β-gals from the microbial sources *Lactobacillus delbrueckii* subsp. *bulgaricus* DSM 20081 (0.919 ± 0.088 mM) [15] and *Paecilomyces aerugineus* (0.65 ± 0.02 mM) [16]. This indicates that Koor β-gal has a lower substrate affinity than the latter two enzymes. However, the catalytic rate (*k*_cat_) of Koor β-gal is higher than that of β-gals from *Lactobacillus delbrueckii* subsp. *bulgaricus* DSM 20081 (603 ± 15 s^−1^) and *Paecilomyces aerugineus* (15.7 s^−1^).

### 2.3. Optimization of GOS Production Conditions

**Figure 3:**
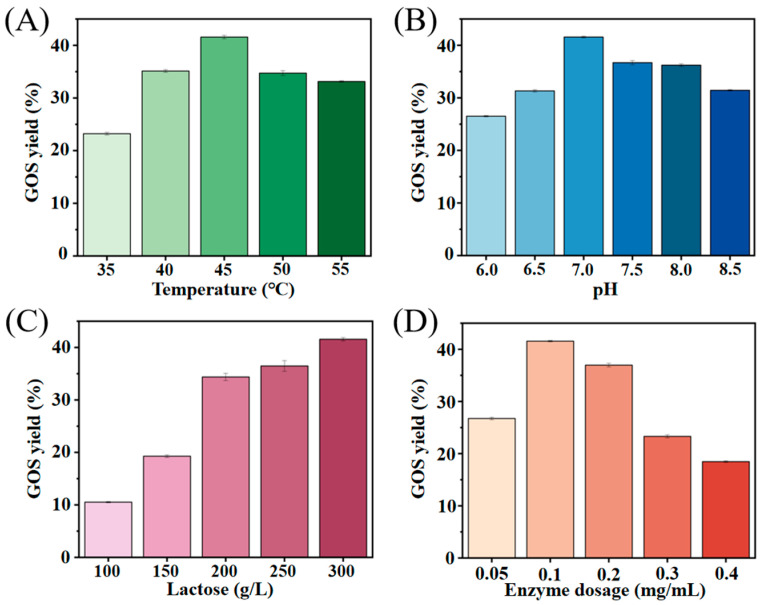
The optimization of GOS production conditions. Effect of temperature (35–55 °C) on GOS yield (**A**), effect of pH (6.0–8.5) on GOS yield (**B**), effect of D-lactose concentration (100–300 g/L) on GOS yield (**C**), and effect of enzyme dosage (0.05–0.4 mg/mL) on GOS yield (**D**). It should be noted that GOS content was calculated by subtracting the amounts of D-glucose, D-galactose, and unreacted D-lactose from the total sugar concentration, and the GOS yield was expressed as the proportion of GOS to total sugars (the content of unreacted D-lactose includes both D-lactose and allolactose; therefore, the term “GOS” here specifically refers to GOS that do not contain allolactose). Error bars represent the SD of three independent biological replicates.

Besides the intrinsic properties of the enzyme, external factors such as temperature, pH, substrate level, and enzyme dosage also played crucial roles in GOS production [13]. As illustrated in Figure 3, optimization was performed across five temperatures (35–55 °C), six pH values (6.0–8.5), D-lactose concentrations ranging from 100 to 300 g/L, and enzyme dosages between 0.05 and 0.4 mg/mL. The highest GOS yield, reaching 41.6%, was obtained under the optimized conditions of 45 °C, pH 7.0, 300 g/L D-lactose, 0.1 g/L enzyme addition and a 13 h reaction time. In comparison to D-lactose concentration and enzyme dosage, temperature and pH had a minimal effect on GOS production, yielding between 31.3% and 41.6% (40–55 °C, pH 6.5–8.5). GOS yield was the lowest at 35 °C (23.2%). The lower GOS yield at this temperature may be partially attributed to the relatively low solubility of D-lactose, which could limit substrate availability for the Koor β-gal catalyzed transglycosylation reaction. This factor together with the relatively low enzyme activity at 35 °C, collectively contributes to the reduced GOS production. Elevating the D-lactose concentration from 100 g/L to 300 g/L resulted in an enhanced yield, increasing from 10.5% to 41.6%. Increasing the enzyme concentration from 0.05 mg/mL to 0.1 mg/mL led to a rise in yield from 26.7% to 41.6%. However, a further increase in enzyme dosage from 0.1 mg/mL to 0.4 mg/mL resulted in a decrease in GOS yield, dropping to 18.5%. It is speculated that increasing the enzyme dosage can accelerate the enzyme-catalyzed reaction, thereby shortening the reaction cycle. However, the catalytically generated products (GOS) are also rapidly hydrolyzed, which is unfavorable for product collection.

Commercial β-gals applied in GOS production are traditionally derived from *Aspergillus niger*, *Bacillus circulans*, and *Kluyveromyces lactis* [17], with typical yields in the range of 30–40% [18]. Advances in biotechnology, however, have enabled the discovery and engineering of β-gals with significantly higher efficiency and GOS yields. For instance, the β-gal from *Paenibacillus antarcticus* exhibited a relatively high reported yield of 50.8% [19]. In a recent study, a β-gal from *Pseudomonas tritici* SWRI145 reported a GOS yield of 44.8% at a D-lactose concentration of 300 g/L [20]. While the β-gal from *Enterobacter cloacae* achieved 51.73% with 380 g/L substrate under the optimal conditions of 40 °C and pH 7.0. Rational design has also generated a mutant variant (H542V) of *E. cloacae* β-gal with a remarkable yield of 67.08% [9]. Moreover, glucose re-tunneling engineering of *B. circulans* produced mutants T215Y and T473Y, which raised yields from the wild-type 52.1% to 57.2% and 57.6%, respectively [21]. Similarly, site-directed mutagenesis of *Sulfolobus solfataricus* β-gal led to variants F411Y, enhancing yields to 61.7% compared with 50.9% for the wild-type enzyme [22].

### 2.4. Products Analysis

**Figure 4:**
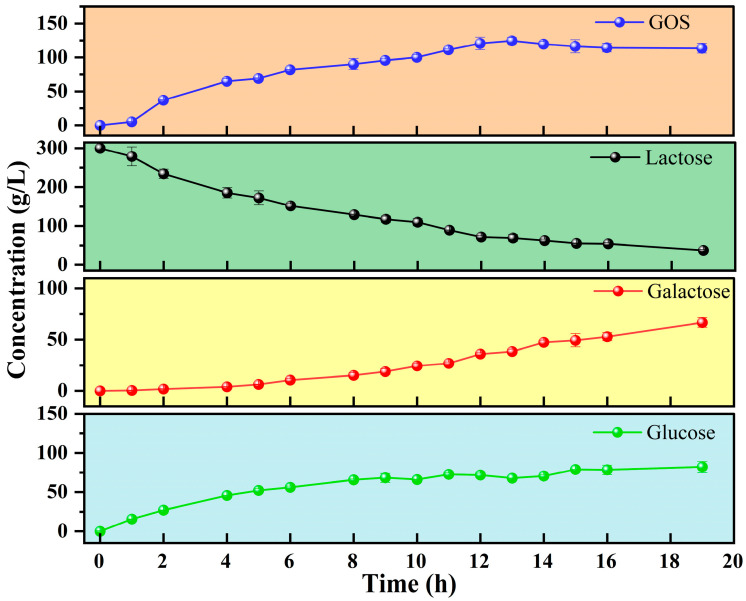
Time courses of enzymatic synthesis of GOS from D-lactose by Koor β-gal, including the concentration of D-lactose (D-lactose and allolactose), GOS, D-galactose, and D-glucose. The reaction was conducted under the following conversion conditions: 300 g/L D-lactose as substrate, 0.1 mg/mL Koor β-gal as catalyst, 1 mM Mg^2+^, pH 7.0, and 45 °C. Error bars represent the SD of three independent biological replicates.

At the optimized conditions for GOS synthesis (45 °C, pH 7.0, 300 g/L D-lactose, and 0.1 g/L enzyme), the reaction progress was tracked by HPLC. As depicted in Figure 4, D-lactose concentration decreased progressively during the reaction, while D-glucose and D-galactose levels rose continuously. The formation of GOS increased over time and reached its maximum after 13 h. After this peak, a slight decline in GOS content was observed. At the point of the highest GOS production, the concentrations of GOS, residual D-lactose (includes both D-lactose and allolactose), D-glucose, and D-galactose were 124.7 g/L, 69.0 g/L, 67.9 g/L, and 38.4 g/L, respectively. As the reaction time was further extended, the D-lactose content further decreased. After 19 h, only 37.4 g/L remained.

**Figure 5:**
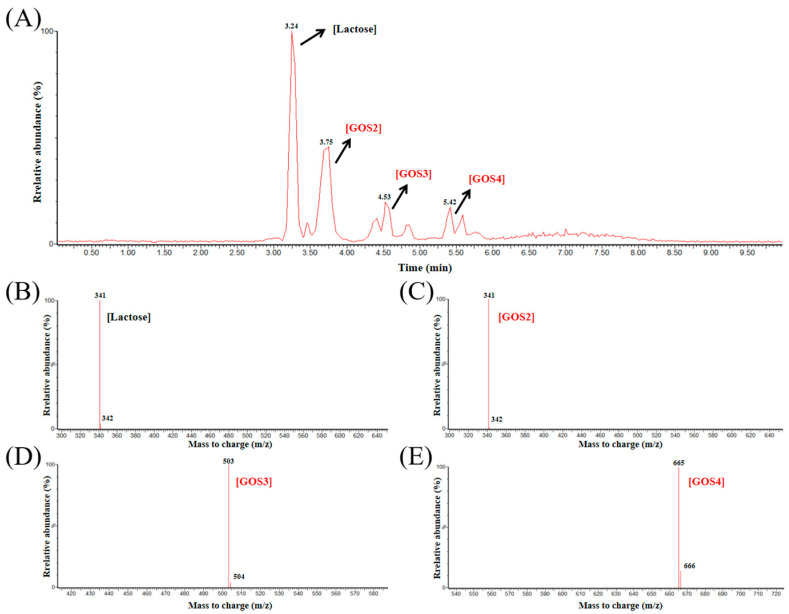
Analysis of Koor β-gal reaction products using LC-MS. (**A**) LC-MS analysis of the products. (**B**–**E**) MS analysis of the products at peaks of 3.24 min (**B**), 3.73 min (**C**), 4.53 min (**D**), and 5.42 min (**E**).

The composition of the synthesized GOS was subsequently analyzed by LC-MS. As shown in Figure 5A, LC analysis displayed four distinct peaks with retention times of 3.24, 3.75, 4.53, and 5.42 min. The subsequent mass spectra (Figure 5B–E) verified the presence of D-lactose and different GOS oligomers. The detected molecular ion signals corresponded to 341.11 *m*/*z* for D-lactose, 341.11 *m*/*z* for GOS2, 503.16 *m*/*z* for GOS3, and 665.22 *m*/*z* for GOS4. These LC-MS results demonstrated that the major products generated by Koor β-gal were GOS with degrees of polymerization (DP) ranging from 2 to 4 (GOS2–GOS4). β-gals from various sources typically yield different types of GOS. For example, β-gal from *Lactobacillus bulgaricus* could efficiently synthesize GOS3 linked by β-(1 → 3) and β-(1 → 4) bonds, achieving a 34% yield [23]. Meanwhile, β-gal from *B. circulans* mainly produced GOS2-GOS5 [21], and β-gal from *Bifidobacterium adolescentis* primarily yielded GOS2-GOS4 [24]. Furthermore, β-gal from *Bifidobacterium bifidum* was capable of producing GOS2-GOS6, exhibiting a strong preference for β-(1 → 3) linkages [25]. However, the linkage types of the synthesized GOS were not determined in our study, which represents a limitation and a direction for future research.

### 2.5. Expression of Koor β-Gal in B. subtilis

*Bacillus subtilis* is widely regarded as a safe and reliable host for the expression of enzymes involved in functional sugar production. In this work, the gene encoding Koor β-gal was inserted into the pP43NMK vector and introduced into *B. subtilis* WB600. Analysis of the fermentation broth revealed that Koor β-gal was secreted into the supernatant, confirming its extracellular expression in *B. subtilis*. After 48 h of flask fermentation, the enzyme activity reached 34.5 U/mL. To further enhance protein expression, optimization strategies were employed, including the selection of suitable expression vectors, promoters, 5′-UTR sequences, and N-terminal coding sequences (NCSs). Specifically, three vectors (pP43NMK, pMA5, and pHT01), ten constitutive promoters (P*_Hpall_*, P*_mmgA_*, P*_odhA_*, P*_phrE_*, P*_sdhB_*, P*_spoVG_*, P*_srfAA_*, P*_yceC_*, P*_yqfD_*, P*_yvyD_*), seven 5′-UTR variants (UTR3, UTR6, UTR7, UTR8, UTR10, UTR11, and UTR13), and ten different NCSs (*Apre NCS+*, *BS-ovalbumin-1*, *De novo8*, *MLD40*, *MLD42*, *MLD47*, *MLD62*, *MLD62-30*, *ydbp*, *ydbp30*) were tested to improve Koor β-gal expression in *B. subtilis*.

**Figure 6:**
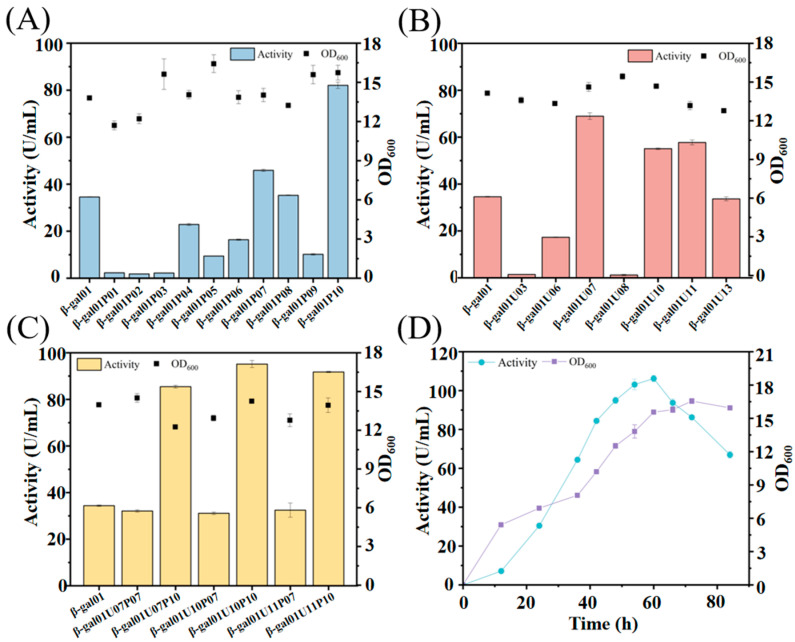
Effects of promoters, 5′-UTR sequences, promoter-5′-UTR synergy, and fermentation duration on Koor β-gal expression activity in *Bacillus subtilis*. (**A**) Effect of different promoters on enzyme activity in culture filtrate and OD_600_. β-gal01: Control strain (pP43NMK vector, P43 promoter); β-gal01P01 -P10: Strains with promoters (P*_Hpall_*, P*_mmgA_*, P*_odhA_*, P*_phrE_*, P*_sdhB_*, P*_spoVG_*, P*_srfAA_*, P*_yceC_*, P*_yqfD_*, P*_yvyD_*) replacing P43. (**B**) Effect of different 5′-UTR on enzyme activity in culture filtrate and OD_600_. β-gal01U03-13: Strains with 5′-UTR variants (UTR3/6/7/8/10/11/13). (**C**) Synergistic effect of optimal promoters (P*_srfAA_*/P*_yvyD_*) and 5′-UTRs (UTR7/10/11) on Koor β-gal activity. (**D**) Shake flask cultivation of strain β-gal01U10P10 for Koor β-gal enzyme expression. Error bars represent the SD of three independent biological replicates.

The results indicated that the optimization of expression vectors (Figure S1) and NCS (Figure S2) did not yield the expected increase in enzyme expression. P*_srfAA_* and P*_yvyD_* among the ten promoters, exhibited enhancements in Koor β-gal expression by 33.0% and 136.6%, resulting in enzyme activity in culture filtrate (48 h) of 45.9 U/mL and 23.8 U/mL, respectively (Figure 6A). Noteworthy enhancements were observed with UTR7, UTR10, and UTR11 among the seven 5′-UTR sequences in Koor β-gal expression. Relative to the control group, the enzyme activity in culture filtrate (48 h) for UTR7, UTR10, and UTR11 reached 69.0 U/mL, 55.1 U/mL, and 57.7 U/mL, with increments of 100%, 59.7%, and 67.2%, respectively (Figure 6B). Furthermore, the synergistic effect of promoter and 5′-UTR on Koor β-gal expression was investigated. Optimal two promoters, P*_srfAA_* and P*_yvyD_*, along with optimal 5′-UTR UTR7, UTR10, and UTR11, were chosen for synergy assessment. Through a comprehensive combination design, six recombinant *B. subtilis* strains were generated (Figure 6C). The strain combining the P*_yvyD_* promoter and UTR10 exhibited the highest Koor β-gal expression level, with an activity of 95.2 U/mL, approximately 176% greater than that of the UTR10 control strain. The influence of fermentation duration in shake-flask cultures on the expression of Koor β-gal was shown in Figure 6D. After 60 h of fermentation, the enzyme activity in culture filtrate reached a maximum of 106.2 U/mL. Despite a slight increase in OD_600_ up to 72 h, the enzyme activity in culture filtrate decreased, suggesting that the optimal fermentation time was 60 h.

At present, only a limited number of reports focused on expressing β-gals in *B. subtilis*. For example, the β-gal from *Bacillus megaterium* YZ08, which was not inhibited by D-galactose or D-glucose, was secreted extracellularly in *B. subtilis*, reaching a peak enzyme activity in culture filtrate of 17.55 U/mL after 72 h [26]. Similarly, the β-gal from *Bacillus aryabhattai* was secreted through a non-classical protein secretion pathway in *B. subtilis*, achieving a maximum activity of 17.41 U/mL [12]. In another study, three copies of Bgal1-3, a β-gal obtained from a marine metagenomic library, were expressed and secreted in *B. subtilis* via both Tat-dependent and Tat-independent pathways, with the enzyme activity in culture filtrate of 2.15 U/mL [27]. In summary, compared with the above-mentioned β-gals expressed in *B. subtilis*, Koor exhibited a higher enzyme activity in culture filtrate.

## 3. Materials and Methods

### 3.1. Plasmid, Strains, and Chemicals

The construction and sequencing of recombinant plasmids were performed by Sangon Biotech Co., Ltd. (Shanghai, China). Host strains, including *E. coli* BL21(DE3) and DH5α, along with D-lactose and other chemical reagents, were supplied by Sinopharm Chemical Reagent Co., Ltd. (Shanghai, China). Standard galactooligosaccharides (GOS) and o-nitrophenyl-β-D-galactopyranoside (ONPG) were purchased from Yuanye Bio-Technology Co., Ltd. (Shanghai, China). The Bradford protein quantification kit was obtained from Beyotime Biotechnology Co., Ltd. (Shanghai, China). Plasmid extraction and gel purification kits were sourced from Tiangen Biotechnology Co., Ltd. (Beijing, China).

### 3.2. Cloning, Expression and Purification of Koor β-Gal

The gene encoding Koor β-gal (NCBI accession number FMAY01000003.1) was chemically synthesized and amplified. It was subsequently inserted into the pET-28a(+) expression vector via NcoI and XhoI restriction enzyme sites. To enable downstream purification, a 6 × His tag was fused to the C-terminus of the gene. The resulting recombinant plasmid was introduced into *E. coli* BL21(DE3) cells for expression. Transformed cells were cultivated in LB medium containing 50 μg/mL kanamycin at 37 °C with shaking at 200 rpm. When cell density reached an OD_600_ of approximately 0.6–0.8, expression was induced using 1 mM IPTG, followed by incubation at 28 °C for 8 h. After induction, the cells were harvested by centrifugation and disrupted by ultrasonic treatment. The recombinant protein containing a C-terminal 6 × His tag was purified using a Ni^2+^-nitrilotriacetic acid (Ni-NTA) affinity chromatography column (1.6 cm × 20 cm). The purification process employed a three-buffer system: a binding buffer consisting of 50 mM HEPES and 500 mM NaCl (pH 7.0), a wash buffer supplemented with an additional 50 mM imidazole, and an elution buffer containing an additional 500 mM imidazole. Under the conditions of the binding buffer, untagged impurities flowed through the column, while the His-tagged protein selectively bound to the resin. Weakly bound contaminants were subsequently removed using the wash buffer. Finally, the target protein was eluted and collected by applying the elution buffer containing 500 mM imidazole. Following purification, the eluted protein was sequentially dialyzed against dialysis buffers to remove residual imidazole and metal ions. The dialysis buffers consisted of 50 mM HEPES buffer, with or without 10 mM EDTA, respectively. Protein purity and concentration of the purified Koor β-gal were evaluated using Bis-Tris SDS-PAGE and a Bradford protein quantification assay.

### 3.3. Enzyme Activity Assay of Koor β-Gal

One unit (U) of enzymatic hydrolytic activity was defined as the quantity of enzyme that hydrolyzes 1 μmol of *o*-nitrophenyl-β-D-galactopyranoside (ONPG) per minute under the following assay conditions [28]. The reaction mixture contained 15 mM ONPG and 10 μg/mL of purified Koor β-gal, and was carried out at pH 7.0 (100 mM HEPES buffer) and 50 °C for 5 min. To terminate the enzymatic reaction, 1 mL of 1 M Na_2_CO_3_ was added. The concentration of *o*-nitrophenol released was quantified by measuring the absorbance at 420 nm using a Tecan Infinite 200 pro microplate spectrophotometer.

### 3.4. Enzymatic Properties Determination

In order to systematically evaluate the biochemical properties, the gene encoding Koor β-gal, which included a C-terminal 6 × His tag, was cloned into the pET-28a(+) vector and expressed in *E. coli* BL21 (DE3). Following IPTG induction, the enzyme was purified using Ni^2+^-affinity chromatography. The optimal pH was determined by assaying the enzyme under the following conditions: 15 mM ONPG as substrate, 10 μg/mL purified Koor β-gal, and 1 mM Mg^2+^ at 50 °C for 5 min in four 100 mM buffer systems—HAC-NaAC (acetic acid-sodium acetate buffer) (pH 5.5–6.0), PBS (pH 6.0–7.0), HEPES (pH 7.0–8.0), and Tris-HCl (pH 8.0–9.0).

For identifying the optimal temperature, reactions were conducted at pH 7.0 within a temperature range of 40–60 °C. Thermostability was assessed by pre-incubating the purified Koor β-gal in 50 mM HEPES buffer (pH 7.0) at temperatures from 35 to 50 °C for defined time periods, followed by measuring the residual activity. Enzyme activity prior to incubation was considered 100% and used as the reference for comparison. To assess the effect of metal ions, 1 mM concentrations of various ions (Na^+^, Zn^2+^, Cu^2+^, Mg^2+^, Co^2+^, Mn^2+^, Ca^2+^, and Ni^2+^) were added to the reaction mixture, and enzyme activity was measured. The kinetic parameters of Koor β-gal toward ONPG were evaluated under standard assay conditions by varying the substrate concentration from 0.3 to 50 mM to calculate kinetic parameters.

### 3.5. Bioconversion of GOS from D-Lactose

To achieve the maximum GOS yield, the reaction conditions were systematically optimized, such as pH, temperature, substrate concentration, and enzyme dosage. The influence of pH was assessed by conducting reactions at 45 °C in buffers ranging from pH 6.5 to 8.5, using a reaction mixture containing 300 g/L D-lactose, 1 mM Mg^2+^, and 0.1 mg/mL of purified Koor β-gal. Temperature optimization was performed under identical conditions within the range of 40 to 55 °C. To evaluate the effect of D-lactose concentration, substrate concentrations were varied between 100 and 300 g/L. Enzyme dosage experiments were carried out by adjusting the concentration of Koor β-gal from 0.05 to 0.4 mg/mL. The reaction time for all these experiments was consistently set at 13 h.

The reaction products were generated using 300 g/L D-lactose as the substrate and 0.1 mg/mL Koor β-gal as the biocatalyst, in the presence of 1 mM Mg^2+^, under conditions of pH 7.0 and 45 °C for a duration of 13 h. Following the reaction, the resulting sugar mixture was analyzed by HPLC to quantify the concentrations of individual sugars [29]. GOS contents were calculated indirectly by subtracting the residual amounts of D-lactose, D-galactose, and D-glucose from the total sugar concentration. It should be noted that the content of “D-lactose” here includes both D-lactose and allolactose. D-Lactose amounts were quantified using HPLC with a refractive index detector (RID) and an XBridge BEH Amide column. D-galactose and D-glucose concentrations were determined with the same HPLC-RID system, but using a Rezex™ ROA-Organic Acid H^+^ column for separation.

To further analyze the composition of oligosaccharides in the reaction products, the liquid chromatograph mass spectrometer (LC-MS) was employed. LC-MS analysis was carried out using a Waters Acquity UPLC system fitted with an ACQUITY UPLC BEH Amide column. The chromatographic separation was achieved using a mobile phase composed of 80% acetonitrile (*v*/*v*), 20% ultrapure water (*v*/*v*), and 0.1% ammonium hydroxide (*v*/*v*). MS detection was performed on a Waters ESI-Q-TOF-MS instrument (Milford, MA, USA) operating in negative electrospray ionization (ESI^−^) mode. Data acquisition was conducted within an *m*/*z* range of 50 to 1500, with the collision energy set at 6/20 eV. During data analysis, the charge state of each detected ion was confirmed as z = −1 (consistent with [M − H]^−^ ions), and this information was included in the identification of D-lactose and GOS.

It should be noted that allolactose, as a disaccharide GOS, is co-eluted with D-lactose under the HPLC conditions used in this study. Therefore, the quantified “residual D-lactose” includes both unreacted D-lactose and any allolactose that may be present. Consequently, the yield of GOS reported in this study specifically refers to GOS that does not contain allolactose.

### 3.6. Expression of Koor β-Gal in B. subtilis

The gene encoding Koor β-gal was inserted into the pP43NMK plasmid under the control of the constitutive P43 promoter, generating the recombinant plasmid pP43NMK-Koor-β-gal. This construction was subsequently introduced into *B. subtilis* WB600. The engineered strain was initially cultured overnight in LB medium containing 50 μg/mL kanamycin at 37 °C with agitation at 200 rpm. For large-scale expression, the overnight culture was transferred into TB medium (24 g/L yeast extract, 12 g/L tryptone, 12.5 g/L K_2_HPO_4_, and 2.3 g/L KH_2_PO_4_) supplemented with 50 μg/mL kanamycin, and incubated at 33 °C with shaking at 200 rpm. Since Koor β-gal was secreted extracellularly in *B. subtilis*, the fermentation broth was centrifuged after cultivation, and the supernatant was used for enzymatic activity assays. In order to further enhance the expression level of Koor β-gal, optimization and regulation were carried out in terms of expression vectors, promoters, 5′-UTR sequences, and N-terminal coding sequences (NCS). Three vectors, namely pP43NMK, pMA5, and pHT01 [30], were utilized for expression vector optimization (Table S2). Ten different constitutive promoters, such as P*_Hpall_*, P*_mmgA_*, P*_odhA_*, P*_phrE_*, P*_sdhB_*, P*_spoVG_*, P*_srfAA_*, P*_yceC_*, P*_yqfD_*, P*_yvyD_* [31], were examined to replace the P43 promoter in pP43NMK harboring Koor β-gal (Table S3). In the optimization of 5′-UTR sequences, previously optimized seven different 5′-UTR sequences (UTR3, UTR6, UTR7, UTR8, UTR10, UTR11, UTR13) [32] were substituted for the native 5′-UTR sequence in the pP43NMK vector (Table S4). For NCS optimization, ten distinct NCSs (*Apre NCS+*, *BS-ovalbumin-1*, *De novo8*, *MLD40*, *MLD42*, *MLD47*, *MLD62*, *MLD62-30*, *ydbp*, *ydbp30*) [33] were appended to the 5′-end of the Koor β-gal gene sequence (Table S5).

## 4. Conclusions

In this study, a novel β-gal from *Kosakonia oryzendophytica* was identified which exhibited a specific activity of 288.6 U/mg towards ONPG. Koor β-gal exhibited good GOS production capabilities, achieving a maximum GOS yield of 41.6%. The primary oligosaccharide products were determined to be GOS2 to GOS4. Additionally, the expression of Koor β-gal in *B. subtilis* was also explored through comprehensive optimization of the promoters and 5′-UTR, with a maximum enzyme activity in culture filtrate of 106.2 U/mL. Collectively, Koor β-gal provided a potential alternative for GOS production.

## Data Availability

All datasets generated for this study are included in the article.

## Supplementary Materials

The following supporting information can be downloaded at: https://www.mdpi.com/article/10.3390/molecules30224343/s1, Figure S1: The enzyme activity in culture filtrate using different vectors. β-gal01 denotes the *B. subtilis* WB600 strain that contains plasmid pP43NMK, which encompasses the P43 promoter and the Koor-encoding gene. β-gal02 denotes the *B. subtilis* WB600 strain that contains plasmid pMA5, which encompasses the P*_HpaII_* promoter and the Koor-encoding gene. β-gal03 denotes the *B. subtilis* WB600 strain that contains plasmid pHT01, which encompasses the P*_grac_* promoter and the Koor-encoding gene. Error bars represent the standard deviation (SD) of three independent biological replicates; Figure S2: The enzyme activity in culture filtrate using different NCSs. β-gal01 denotes the *B. subtilis* WB600 strain that contains plasmid pP43NMK, which encompasses the P43 promoter and the Koor-encoding gene. β-gal01N01 to β-gal01N10 represent the variants generated by incorporating *Apre NCS+*, *BS-ovalbumin-1*, *De novo8*, *MLD40*, *MLD42*, *MLD47*, *MLD62*, *MLD62-30*, *ydbp*, *ydbp30*, respectively, into the plasmid of β-gal01. Error bars represent the standard deviation (SD) of three independent biological replicates; Figure S3: The influence of the metal ion Mg^2+^ on the thermostability of Koor β-gal. Error bars represent the standard deviation (SD) of three independent biological replicates; Figure S4: Substrate inhibition kinetic curve of Koor β-gal. The *V* was measured at varying concentrations of ONPG. Error bars represent the standard deviation (SD) of three independent biological replicates; Table S1: *B. sutbilis* WB600 strains used in this study; Table S2: The nucleotide sequences of all NCSs used in this study; Table S3: The nucleotide sequences of all 5′-UTR sequences used in this study; Table S4: The sequences of various promoters; Table S5: Primers used for constructing recombinant plasmids.
