# Supplementary material for: Characterization of a β-Galactosidase from Kosakonia oryzendophytica and Its Heterologous Expression in Bacillus subtilis for Galactooligosaccharides Production
Source: Molecules. 2025 Nov 10;30(22):4343. doi: 10.3390/molecules30224343 (PMC12654650; doi:10.3390/molecules30224343)
Supplement: Supplementary file 1 [file molecules-30-04343-s001.zip › molecules-3893568-supplementary.pdf]

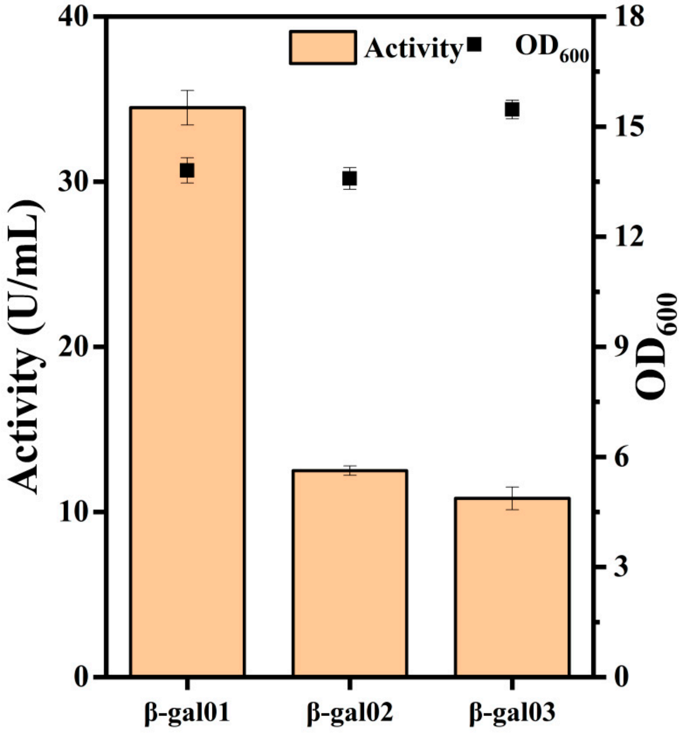

Figure S1. The enzyme activity in culture filtrate using different vectors.

β-gal01 denotes the *B. subtilis* WB600 strain that contains plasmid pP43NMK, which encompasses the P43 promoter and the Koor-encoding gene. β-gal02 denotes the *B. subtilis* WB600 strain that contains plasmid pMA5, which encompasses the P_{HpaII} promoter and the Koor-encoding gene. β-gal03 denotes the *B. subtilis* WB600 strain that contains plasmid pHT01, which encompasses the P_{grac} promoter and the Koor-encoding gene. Error bars represent the standard deviation (SD) of three independent biological replicates.

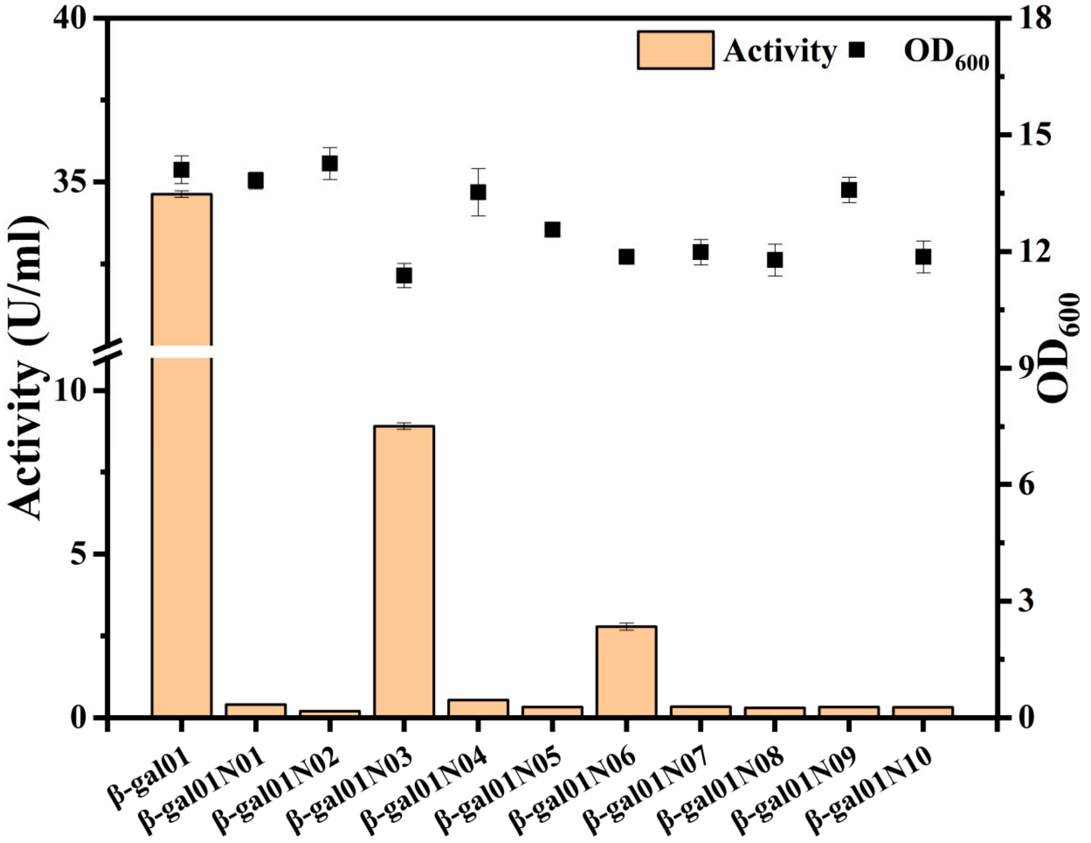

Figure S2. The enzyme activity in culture filtrate using different NCSs.

β-gal01 denotes the *B. subtilis* WB600 strain that contains plasmid pP43NMK, which encompasses the P43 promoter and the Koor-encoding gene. β-gal01N01 to β-gal01N10 represent the variants generated by incorporating *Apre NCS+, BS-ovalbumin-1, De novo8, MLD40, MLD42, MLD47, MLD62, MLD62-30, ydbp, ydbp30*, respectively, into the plasmid of β-gal01. Error bars represent the standard deviation (SD) of three independent biological replicates.

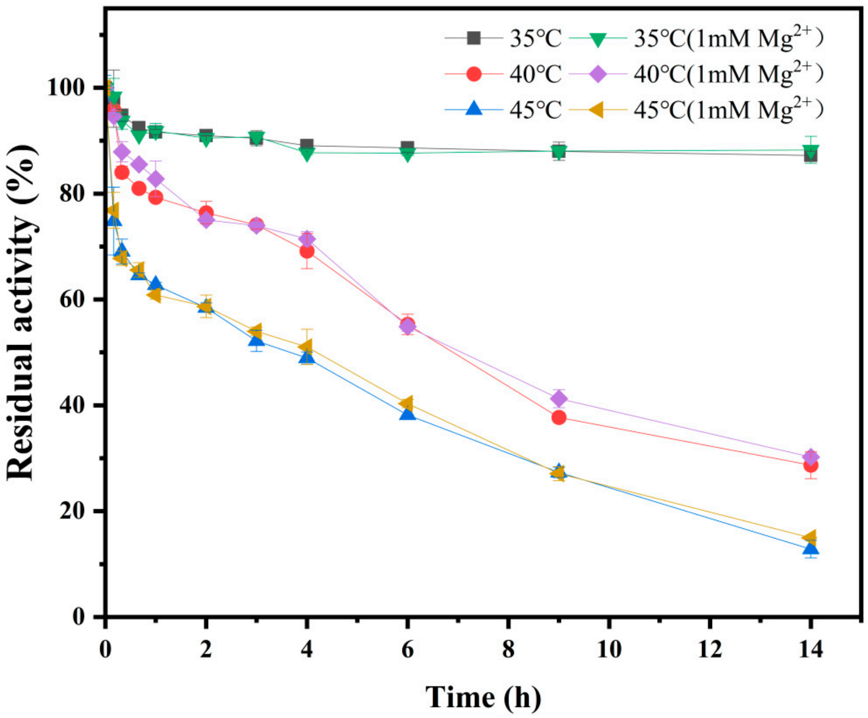

Figure S3. The influence of the metal ion Mg^{2+}on the thermostability of Koor β-gal.

Error bars represent the standard deviation (SD) of three independent biological replicates.

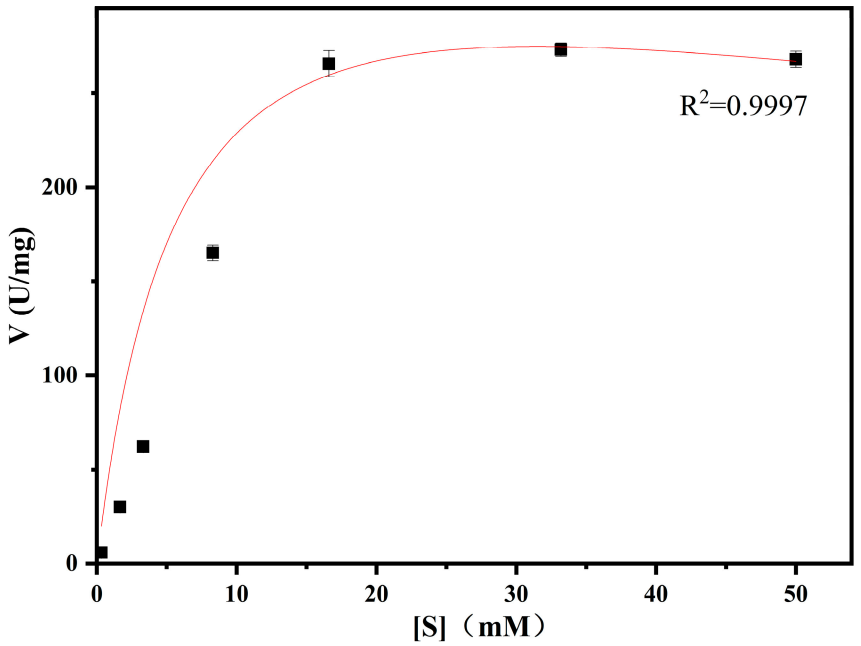

Figure S4. Substrate inhibition kinetic curve of Koor β-gal.

The *V* was measured at varying concentrations of ONPG. Error bars represent the standard deviation (SD) of three independent biological replicates.

Table S1. *B.sutbilis* WB600 strains used in this study.

Strain	Characteristics
β-gal01	WB600 harboring plasmid pP43NMK harboring P43 and Koor encoding gene
β-gal02	WB600 harboring plasmid pMA5 harboring P_{HpaII} and Koor encoding gene
β-gal03	WB600 harboring plasmid pHT01 harboring P_{grac} and Koor encoding gene
β-gal01N01	WB600 harboring plasmid pP43NMK harboring P43, *Apre NCS+* and Koor encoding gene
β-gal01N02	WB600 harboring plasmid pP43NMK harboring P43, *BS-ovalbumin-1* and Koor encoding gene
β-gal01N03	WB600 harboring plasmid pP43NMK harboring P43, *De novo8* and Koor encoding gene
β-gal01N04	WB600 harboring plasmid pP43NMK harboring P43, *MLD40* and Koor encoding gene
β-gal01N05	WB600 harboring plasmid pP43NMK harboring P43, *MLD42* and Koor encoding gene
β-gal01N06	WB600 harboring plasmid pP43NMK harboring P43, *MLD47* and Koor encoding gene
β-gal01N07	WB600 harboring plasmid pP43NMK harboring P43, *MLD62* and Koor encoding gene
β-gal01N08	WB600 harboring plasmid pP43NMK harboring P43, *MLD62-30* and Koor encoding gene
β-gal01N09	WB600 harboring plasmid pP43NMK harboring P43, *ydbp* and Koor encoding gene
β-gal01N10	WB600 harboring plasmid pP43NMK harboring P43, *ydbp30* and Koor encoding gene
β-gal01U03	WB600 harboring plasmid pP43NMK harboring P43, UTR3 and Koor encoding gene
β-gal01U06	WB600 harboring plasmid pP43NMK harboring P43, UTR6 and Koor encoding gene
β-gal01U07	WB600 harboring plasmid pP43NMK harboring P43, UTR7 and Koor encoding gene
β-gal01U08	WB600 harboring plasmid pP43NMK harboring P43, UTR8 and Koor encoding gene
β-gal01U10	WB600 harboring plasmid pP43NMK harboring P43, UTR10 and Koor encoding gene
β-gal01U11	WB600 harboring plasmid pP43NMK harboring P43, UTR11 and Koor encoding gene
β-gal01U13	WB600 harboring plasmid pP43NMK harboring P43, UTR13 and Koor encoding gene

β-gal01P01	WB600 harboring plasmid pP43NMK harboring P_{HpaII} and Koor encoding gene
β-gal01P02	WB600 harboring plasmid pP43NMK harboring P_{mmgA} and Koor encoding gene
β-gal01P03	WB600 harboring plasmid pP43NMK harboring P_{odhA} and Koor encoding gene
β-gal01P04	WB600 harboring plasmid pP43NMK harboring P_{phrE} and Koor encoding gene
β-gal01P05	WB600 harboring plasmid pP43NMK harboring P_{sdhB} and Koor encoding gene
β-gal01P06	WB600 harboring plasmid pP43NMK harboring P_{spoVG} and Koor encoding gene
β-gal01P07	WB600 harboring plasmid pP43NMK harboring P_{srfAA} and Koor encoding gene
β-gal01P08	WB600 harboring plasmid pP43NMK harboring P_{yceC} and Koor encoding gene
β-gal01P09	WB600 harboring plasmid pP43NMK harboring P_{yqfD} and Koor encoding gene
β-gal01P10	WB600 harboring plasmid pP43NMK harboring P_{yvyD} and Koor encoding gene
β-gal01U07P07	WB600 harboring plasmid pP43NMK harboring UTR7 and P_{srfAA} Koor encoding gene
β-gal01U07P10	WB600 harboring plasmid pP43NMK harboring UTR7 and P_{yvyD} Koor encoding gene
β-gal01U10P07	WB600 harboring plasmid pP43NMK harboring UTR10 and P_{srfAA} Koor encoding gene
β-gal01U10P10	WB600 harboring plasmid pP43NMK harboring UTR10 and P_{yvyD} Koor encoding gene
β-gal01U11P07	WB600 harboring plasmid pP43NMK harboring UTR13 and P_{srfAA} Koor encoding gene
β-gal01U11P10	WB600 harboring plasmid pP43NMK harboring UTR13 and P_{yvyD} Koor encoding gene

Table S2. The nucleotide sequences of all NCSs sequences used in this study.

**NCSs**	**Sequence（5′ → 3′）**
Apre NCS+	AGAAGTAAAAAATTATGGATAAGTTTATTA
BS-ovalbumin-1	GATAAGCTAAGAGGCCGTCGACGGTCTAGC
De novo8	ATGAACAACATCAAAAAAGTAGAAGAAACAAACAACCA AAAAAAA
MLD40	ATGAAAAAAATCACAAATAACAGGCAAAACCAAACACT GAAACAA
MLD42	ATGAAAAAAAAAACAACAAACAGGCAAAACCAAAATCT GAAACAA
MLD47	ATGAAAAAAATCACAACAAACAAACAAAACAATGAAGT CAAACAG
MLD62	ATGAAAAAAATCACAAACAACAGGCAAAACCAAACACT GAAAGGT
MLD62-30	ATGAAAAAAATCACAAACAACAGGCAAAAC
ydbp	ATGAAAAAAATCACAACAAACGAACAATTTAATGAACT GATTCAA
ydbp30	ATGAAAAAAATCACAACAAACGAACAATTT

Table S3. The nucleotide sequences of all 5′-UTR sequences used in this study.

**5′-UTR**	**Sequence（5′ → 3′）**
Native	GTGATAGCGGTACCATTATAGGTAAGAGAGGAATGTACAC
UTR3	GTGATTAGAAAGGAGGAATGTACAC
UTR6	GTGATAGCGGTACATTAGAAAGGAGGAATGTACA
UTR7	GTGATAGCGGTACATTAGAAAGGAGGAATGTATA
UTR8	GTGATAGCGGTATATTAGAAAGGAGGAATGTATA
UTR10	GTACATTAGAAAGGAGGAATGTACA
UTR11	GTACATTAGAAAGGAGGAATGTATA
UTR13	GTGATTAGAAAGGAGGAATGTACA

Table S4. The sequences of various promoters.

**Promoters**	**Sequence（5′ → 3′）**
P43	TGATAGGTGGTATGTTTTCGCTTGAACTTTTAAATACAGCCA<br>TTGAACATACGGTTGATTTAATAACTGACAAACATCACCCTC<br>TTGCTAAAGCGGCCAAGGACGCTGCCGCCGGGGCTGTTTGC<br>GTTTTTGCCGTGATTTCGTGTATCATTGGTTTACTTATTTTT<br>TGCCAAAGCTGTAATGGCTGAAAATTCTTACATTTATTTTAC<br>ATTTTTAGAAATGGGCGTGAAAAAAAGCGCGCGATTATGTA<br>AAATATAAAGTGATAGCGGTACCATTATAG
P_{HpaII}	GATCTTCTCAAAAAATACTACCTGTCCCTTGCTGATTTTTAA<br>ACGAGCACGAGAGCAAAACCCCCCTTTGCTGAGGTGGCAGA<br>GGGCAGGTTTTTTTGTTTCTTTTTTCTCGTAAAAAAAAGAAA<br>GGTCTTAAAGGTTTTATGGTTTTGGTCGGCACTGCCGCGCCT<br>CGCAGAGCACACACTTTATGAATATAAAGTATAGTGTGTTAT<br>ACTTTACTTGGAAGTGGTTGCCGGAAAGAGCGAAAATGCCT<br>CACATTTGTGCCACCTAA
P_{mmgA}	TCGATATATCGCGTCTATTCCGGCTTCCGGCTATCACCCGAA<br>GATAAACAGCCCAGGGGTCACAGATGAAGTACTGAAGAAA<br>ATGAGGAACGGTTTGATTAAGGTAAGGCCGTATACAGTCAA<br>TCGTCCGGAAGATATGAAGCGTCTCATTGAAGCGGGAGCAG<br>ACGGCATGTTTACCGACTTTCCAGAAAAGGCTTCGGCATTGC<br>TGAAAAATGAATAGTTGTTAGAAGGAGGCTGTTTGACGCAG<br>CCTTCTTTTTTCATTCATTCATGCCCGTTTCAAAGCATACATT<br>CATAGAAGAC
P_{odhA}	GAATAGAAGTGATAAATGTTTTTCTCTCCAACTCAGAAATAG<br>AAGAATCGCAAATGAAGACGATCCAGGATATGTACGGAAA<br>ATTCAGTATATTTGTCCCGGATGTTGATCAGCTACCGGATGT<br>TTTGTATCCGCTGCTGAAAAAACTGCTTCATAAAAGCATAGG<br>ATAGCCCTTAATCCTATGCTTTTTGGCGTTTGTTTTTTCGAAT<br>GATTAAATTTTTTGTTTTTTATAAAGGTTTTTTACTATTTTGT<br>GAACAATCAAGGTAGAATCAAATTGCAAACAGTGGTAAAAT<br>ATCGTTG
P_{phrE}	CGTTGATTTATTTTAAACAAGGCAAAAAAGAACAAGCAATG<br>GATTGCTTCCGCAAAGGAATCAGAAGTGCTGTAGATTTTAA<br>AGACGAACTATTTATGAACTTATTTGAAGCATTAGACGTTCT<br>TTATATAAGAAATGGCGATACACCTAAACTTCTCAACATTTT<br>TTCTCGTTTAGAGAACGGCAAAGGATACCCTTATCTGGAAG<br>AGCTGGCATTGTTAGGAGGCAACCTTTTCGATTATAATGGAA<br>AAATAGAGGATAGTATCATCTGTTTCAAGAAGATGGTATAT<br>GCTCAAAAGC
P_{sdhB}	AATTTCTTTTAGGTTGTCAATAGATGCTTCAAATTTAGACTT<br>CATATCTGTCTCCCCTCTCTCCTGCGTATATTATAGCTCAAAC<br>AGGGGGGAGGATTACAGAATGATCCTGTAATTCTTATGAAA

	AATTAAGCAAGAAATATATATTGATAAAATAAAATTTTTCA<br>ATCAACTAATCAATTCGGAAAATTATAATTTATGTACGCGTT<br>TTCTTGACGCCCTTTTGAGGGAGGAGTAAAATGAAATTGTCA<br>ATAAATCTTAATAAAGTGCTTACAATTGAAAGAAGTGGGGG<br>AAGAGATT
P_{spoVG}	TGCGGAAGTAAACGAAGTGTACGGACAATATTTTGACACTC<br>ACAAACCGGCGAGATCTTGTGTTGAAGTCGCGAGACTCCCG<br>AAGGATGCGTTAGTCGAGATCGAAGTTATTGCACTGGTGAA<br>ATAATAAGAAAAGTGATTCTGGGAGAGCCGGGATCACTTTT<br>TTATTTACCTTATGCCCGAAATGAAAGCTTTATGACCTAATT<br>GTGTAACTATATCCTATTTTTTCAAAAAATATTTTAAAAACG<br>AGCAGGATTTCAGAAAAAATCGTGGAATTGATACACTAATG<br>CTTTTATATAG
P_{srfAA}	GACGCTCTTCGCAAGGGTGTCTTTTTTTGCCTTTTTTTCGGTT<br>TTTGCGCGGTACACATAGTCATGTAAAGATTGTAAATTGCAT<br>TCAGCAATAAAAAAAGATTGAACGCAGCAGTTTGGTTTAAA<br>AATTTTTATTTTTCTGTAAATAATGTTTAGTGGAAATGATTG<br>CGGCATCCCGCAAAAAATATTGCTGTAAATAAACTGGAATC<br>TTTCGGCATCCCGCATGAAACTTTTCACCCATTTTTCGGTGA<br>TAAAAACATTTTTTTCATTTAAACTGAACGGTAGAAAGATAA<br>AAAATAT
P_{yceC}	TTCTCCTTTAGAAACAGGTGCCTGATCAAGTATGCTTAAATG<br>AATCAAAAGGCCGCTTCCTTTCTGTCTGTTTTTAGATGGTGA<br>CACCAGAATAGGCAAAATGACTTGCCCGCGTCAAATGGTTT<br>GTTTAAGAAGCGCACTTTATAGCATTTGCAAATCATATATCA<br>AATATGGAAGTTTTTTTTCGAGGTTTTTCGTCAATTATTCTTA<br>ACTTTTACGAAACTTTGATATAATAACAAACGTATATATTAG<br>TAATTTACGGCTTATTTTCCTTGTGAGCGTAAAAATAAATGT<br>GACTAT
P_{yqfD}	AGATGCCCCTGCACCTATCCCTAACCGTATGGAACAGGCAA<br>GACGGGAAGCGGAAGAAAGACGCAGGGAAACAGCAAGAAA<br>CCTGAAAGGGCTGGAACGAGATCTTGCTGCTGCCAAACAAA<br>AAACAGTATACACAAAACAAAAAATGCTTCAGGTGAATAAA<br>GACACCGTCGTACAGGGGATCGTTCTAGGAGAGGTGTTCGG<br>ACCTCCACGGGCGAAAAAACCTCACCGTACGATGCGCCCGG<br>CCCGTAAAAATTAAAGTGTTAGAACCTCCTTTCAAATCATAC<br>ATATGAGATGAAA
P_{yvyD}	GATCAATTGGTCTCTTTCTCTTTTCCCTCTCATGAGTTCTGTG<br>AGTATTTAAAGGAACATTTTCTGATTCATTATAGAAAATGGA<br>TGCTGTCTATTCATCAATGTATGGAACCCTTTTTAATCAATT<br>AGGCGTGTGTGAGGTATTTGTTTCGTTCAATCAGCATATACA<br>TATACCTCCGAACCGCCAATAACAGAGCAAATACAAACAAA<br>ATTCGACAAAGTTCACTGAATTTTCACAAAAGATTTATGTTT

CAGCAGGAATTGTAAAGGGTAAAAGAGAAATAGATACATAT
CCTTAAT

Table S5. Primers used for constructing recombinant plasmids.

Plasmids	Primers	Sequence（5′ → 3′）
β-gal01	β-gal01-P-F	GAATGTACACATGTCTGCTTCCGCATTACGTACGTTG
	β-gal01-P-R	CTTTCATCATCAGTCCTGCCGCCAGCAAAAGGCGTAAC
	β-gal01-V-F	CAGGACTGATGATGAAAGCTTGGCGTAATCATGGTCATAGCTG
	β-gal01-V-R	GAAGCAGACATGTGTACATTCCTCTCTTACCTATAATGGTACCGCTATCAC
β-gal02	β-gal02-P-F	CCATGTCTGCTTCCGCATTACGTACGTTGATTTCCCGCCGTG
	β-gal02-P-R	CCGGGTCAGTCCTGCCGCCAGCAAAAGGCGTAAC
	β-gal02-V-F	GCAGGACTGACCCGGGGCAGCCCGCCTAATGAG
	β-gal02-V-R	GTAATGCGGAAGCAGACATGGATCCTTCCTCCTTTAATTGGGAATTGTTATCCGC
β-gal03	β-gal03-P-F	CATATGATGTCTGCTTCCGCATTACGTACGTTGATTTCCCGCCGTG
	β-gal03-P-R	CTAGAGGATCCTCAGTCCTGCCGCCAGCAAAAGGCGTAAC
	β-gal03-V-F	GCAGGACTGAGGATCCTCTAGAGTCGAGCTCAAGCTAGCTTGGTACG
	β-gal03-V-R	GCGGAAGCAGACATCATATGTAAATCGCTCCTTTTTAGGTGGCACAAATG
β-gal01N01	β-gal01N01-F	GTAAAAAATTATGGATAAGTTTATTAATGTCTGCTTCCGCATTACGTACGTTG
	β-gal01N01-R	AACTTATCCATAATTTTTTACTTCTGTGTACATTCCTCTCTTACCTATAATGGTACCGCTATCAC
β-gal01N02	β-gal01N02-F	GCTAAGAGGCCGTCGACGGTCTAGCATGTCTGCTTCCGCATTACGTACGTTG
	β-gal01N02-R	ACCGTCGACGGCCTCTTAGCTTATCGTGTACATTCCTCTCTTACCTATAATGGTACCGC
β-gal01N03	β-gal01N03-F	AACAACAAACAGGCAAAACCAAAATCTGAAACAAATGTCTGCTTCCGCATTACGTACGTTG
	β-gal01N03-R	GGTTTTGCCTGTTTGTTGTTTTTTTTTCATGTGTACATTCCTCTCTTACCTATAATGGTACCGC
β-gal01N04	β-gal01N04-F	CAAAAAAGTAGAAGAAACAAACAACCAAAAAAAAATGTCTGCTTCCGCATTACGTACGTTG
	β-gal01N04-R	TTGTTTCTTCTACTTTTTTGATGTTGTTCATGTGTACATTCCTCTCTTACCTATAATGGTACCGC
β-gal01N05	β-gal01N05-F	CACAAATAACAGGCAAAACCAAACACTGAAACAAATGTCTGCTTCCGCATTACGTACGTTG
	β-gal01N05-R	GGTTTTGCCTGTTATTTGTGATTTTTTTCATGTGTACATTCCTCTCTTACCTATAATGGTACCGC
β-gal01N06	β-gal01N06-F	CACAACAAACGAACAATTTAATGAACTGATTCAAATGTCTGCTTCCGCATTACGTACGTTG

	β-gal01N06-R	TAAATTGTTCGTTTGTTGTGATTTTTTTCATGTGTACATTCCTCTCTTACCTATAATGGTACCGCTATCAC
β-gal01N07	β-gal01N07-F	CACAACAAACAAACAAAACAATGAAGTCAAACAGATGTCTGCTTCCGCATTACGTACGTTG
	β-gal01N07-R	TGTTTTGTTTGTTTGTTGTGATTTTTTTCATGTGTACATTCCTCTCTTACCTATAATGGTACCGC
β-gal01N08	β-gal01N08-F	CAAACAACAGGCAAAACCAAACACTGAAAGGTATGTCTGCTTCCGCATTACGTACGTTG
	β-gal01N08-R	GGTTTTGCCTGTTGTTTGTGATTTTTTTCATGTGTACATTCCTCTCTTACCTATAATGGTACCGC
β-gal01N09	β-gal01N09-F	GAAAAAAATCACAAACAACAGGCAAAACATGTCTGCTTCCGCATTACGTACGTTG
	β-gal01N09-R	TGTTGTTTGTGATTTTTTTCATGTGTACATTCCTCTCTTACCTATAATGGTACCGC
β-gal01N10	β-gal01N10-F	GAAAAAAATCACAACAAACGAACAATTTATGTCTGCTTCCGCATTACGTACGTTG
	β-gal01N10-R	CGTTTGTTGTGATTTTTTTCATGTGTACATTCCTCTCTTACCTATAATGGTACCGCTATCAC
β-gal01U03	β-gal01U03-F	GATTAGAAAGGAGGAATGTACACATGTCTGCTTCCGCATTACGTACGTTG
	β-gal01U03-R	GTACATTCCTCCTTTCTAATCACTTTATATTTTACATAATCGCGCGCTTTTTTTCACGCC
β-gal01U06	β-gal01U06-F	GAAAGGAGGAATGTACAATGTCTGCTTCCGCATTACGTACG
	β-gal01U06-R	CATTGTACATTCCTCCTTTCTAATGTACCGCTATCACTTTATATTTTACATAATCGCG
β-gal01U07	β-gal01U07-F	GAAAGGAGGAATGTATAATGTCTGCTTCCGCATTACGTACG
	β-gal01U07-R	CATTATACATTCCTCCTTTCTAATGTACCGCTATCACTTTATATTTTACATAATCGCG
β-gal01U08	β-gal01U08-F	GAAAGGAGGAATGTATAATGTCTGCTTCCGCATTACGTACG
	β-gal01U08-R	CATTATACATTCCTCCTTTCTAATATACCGCTATCACTTTATATTTTACATAATCGCG
β-gal01U10	β-gal01U10-F	CATTAGAAAGGAGGAATGTACAATGTCTGCTTCCGCATTACGTACG
	β-gal01U10-R	ACATTCCTCCTTTCTAATGTACTTTATATTTTACATAATCGCGCGCTTTTTTTCACGCC
β-gal01U11	β-gal01U11-F	CATTAGAAAGGAGGAATGTATAATGTCTGCTTCCGCATTACGTACG
	β-gal01U11-R	ACATTCCTCCTTTCTAATGTACTTTATATTTTACATAATCGCGCGCTTTTTTTCACGCC
β-gal01U13	β-gal01U13-F	GATTAGAAAGGAGGAATGTACAATGTCTGCTTCCGCATTACGTACGTTG
	β-gal01U13-R	GTACATTCCTCCTTTCTAATCACTTTATATTTTACATAATCGCGCGCTTTTTTTCACGCC
β-gal01P01	β-gal01P01-F	ATTTTTTTGAGCAACTGGATCCGATCTTCTCAAAAAATACTACCTGTCCCTTGCTG
	β-gal01P01-R	ATGTGTACATTCCTCTCTTACTTAGGTGGCACAAATGTGAGGCATTTTC

β-gal01P02	β-gal01P02-F	TTTTTTGAGCAACTGGATCCTCGATATATCGCGTCTATTCCGGCTTCCG
	β-gal01P02-R	ATGTGTACATTCCTCTCTTACGTCTTCTATGAATGTATGCTTTGAAACGGGCATG
β-gal01P03	β-gal01P03-F	TTTTTTGAGCAACTGGATCCGAATAGAAGTGATAAATGTTTTTCTCTCCAACTCAGAAATAGAAGAATCG
	β-gal01P03-R	ATGTGTACATTCCTCTCTTACCAACGATATTTTACCACTGTTTGCAATTTGATTCTACCTTG
β-gal01P04	β-gal01P04-F	TTTTTTGAGCAACTGGATCCCGTTGATTTATTTTAAACAAGGCAAAAAAGAACAAGCAATG
	β-gal01P04-R	ATGTGTACATTCCTCTCTTACGCTTTTGAGCATATACCATCTTCTTGAAACAGATGATAC
β-gal01P05	β-gal01P05-F	TTTTTTTGAGCAACTGGATCCAATTTCTTTTAGGTTGTCAATAGATGCTTCAAATTTAGACTTCATATCTG
	β-gal01P05-R	ATGTGTACATTCCTCTCTTACAATCTCTTCCCCCACTTCTTTCAATTGTAAGCAC
β-gal01P06	β-gal01P06-F	TTTTTTGAGCAACTGGATCCTGCGGAAGTAAACGAAGTGTACGGAC
	β-gal01P06-R	ATGTGTACATTCCTCTCTTACCTATATAAAAGCATTAGTGTATCAATTCCACGATTTTTTCTGAAATCCTG
β-gal01P07	β-gal01P07-F	TTTTTTGAGCAACTGGATCCGACGCTCTTCGCAAGGGTGTCTTTTTTTG
	β-gal01P07-R	ATGTGTACATTCCTCTCTTACATATTTTTTATCTTTCTACCGTTCAGTTTAAATGAAAAAAATGTTTTTATC
β-gal01P08	β-gal01P08-F	TTTTTTGAGCAACTGGATCCTTCTCCTTTAGAAACAGGTGCCTGATCAAGTATG
	β-gal01P08-R	ATGTGTACATTCCTCTCTTACATAGTCACATTTATTTTTACGCTCACAAGGAAAATAAGCC
β-gal01P09	β-gal01P09-F	TTTTTTGAGCAACTGGATCCAGATGCCCCTGCACCTATCCCTAAC
	β-gal01P09-R	ATGTGTACATTCCTCTCTTACTTTCATCTCATATGTATGATTTGAAAGGAGGTTCTAACACTTTAATTTTTACGGGCCG
β-gal01P10	β-gal01P10-F	TTTTTTGAGCAACTGGATCCGATCAATTGGTCTCTTTCTCTTTTCCCTCTCATGAG
	β-gal01P10-R	ATGTGTACATTCCTCTCTTACATTAAGGATATGTATCTATTTCTCTTTTACCCTTTACAATTCCTGCTG
Vector primer	p43NMK-β-gal-F	GTAAGAGAGGAATGTACACATGTCTGCTTCCGCATTACGTACGTTG
	p43NMK-β-gal-R	GGATCCAGTTGCTCAAAAAAATCTCGGTCAGATGTTAC
β-gal01U07P07	β-gal01U07P07-F	GATAGCGGTACATTAGAAAGGAGGAATGTATAATGTCTGCTTCCGCATTACGTACGTTG

	β-gal01U07P07-R	CTTTCTAATGTACCGCTATCACATATTTTTTATCTTTCTACCGTTCAGTTTAAATGAAAAAAATGTTTTTATCACC
β-gal01U07P10	β-gal01U07P10-F	GATAGCGGTACATTAGAAAGGAGGAATGTATAATGTCTGCTTCCGCATTACGTACGTTG
	β-gal01U07P10-R	CTTTCTAATGTACCGCTATCACATTAAGGATATGTATCTATTTCTCTTTTACCCTTTACAATTCCTGCTG
β-gal01U10P07	β-gal01U10P07-F	CATTAGAAAGGAGGAATGTACAATGTCTGCTTCCGCATTACGTACGTTG
	β-gal01U10P07-R	GTACATTCCTCCTTTCTAATGTACATATTTTTTATCTTTCTACCGTTCAGTTTAAATGAAAAAAATGTTTTTATC
β-gal01U10P10	β-gal01U10P10-F	CATTAGAAAGGAGGAATGTACAATGTCTGCTTCCGCATTACGTACGTTG
	β-gal01U10P10-R	GTACATTCCTCCTTTCTAATGTACATTAAGGATATGTATCTATTTCTCTTTTACCCTTTACAATTCCTGCTG
β-gal01U11P07	β-gal01U11P07-F	GTACATTAGAAAGGAGGAATGTATAATGTCTGCTTCCGCATTACGTACGTTG
	β-gal01U11P07-R	CATTCCTCCTTTCTAATGTACATATTTTTTATCTTTCTACCGTTCAGTTTAAATGAAAAAAATGTTTTTATCACC
β-gal01U11P10	β-gal01U11P10-F	GTACATTAGAAAGGAGGAATGTATAATGTCTGCTTCCGCATTACGTACGTTG
	β-gal01U11P10-R	CATTCCTCCTTTCTAATGTACATTAAGGATATGTATCTATTTCTCTTTTACCCTTTACAATTCCTGCTG
